# The CHICO (Children's Cough) Trial protocol: a feasibility randomised controlled trial investigating the clinical and cost-effectiveness of a complex intervention to improve the management of children presenting to primary care with acute respiratory tract infection

**DOI:** 10.1136/bmjopen-2015-008615

**Published:** 2015-09-15

**Authors:** Sophie L Turnbull, Niamh M Redmond, Patricia Lucas, Christie Cabral, Jenny Ingram, Sandra Hollinghurst, Alastair D Hay, Tim J Peters, Jeremy Horwood, Paul Little, Nick Francis, Peter S Blair

**Affiliations:** 1 Centre for Academic Primary Care, School of Social and Community Medicine, University of Bristol, Bristol, UK; 2 School for Policy Studies, University of Bristol, Bristol, UK; 3 School of Social and Community Medicine, University of Bristol, Bristol, UK; 4 School of Social and Community Medicine, Centre for Child and Adolescent Health, Bristol, UK; 5 School of Clinical Sciences, University of Bristol, Bristol, UK; 6 Department of Primary Care & Population Sciences, University of Southampton, Aldermoor Health Centre, Southampton, UK; 7 Institute of Primary Care and Public Health, University of Cardiff, Cardiff, UK

**Keywords:** PRIMARY CARE, RESPIRATORY MEDICINE (see Thoracic Medicine)

## Abstract

**Introduction:**

While most respiratory tract infections (RTIs) will resolve without treatment, many children will receive antibiotics and some will develop severe symptoms requiring hospitalisation. There have been calls for evidence to reduce uncertainty regarding the identification of children who will and will not benefit from antibiotics. The aim of this feasibility trial is to test recruitment and the acceptance of a complex behavioural intervention designed to reduce antibiotic prescribing, and to inform how best to conduct a larger trial.

**Methods and analysis:**

The CHICO (Children's Cough) trial is a single-centre feasibility cluster randomised controlled trial (RCT) comparing a web-based, within-consultation, behavioural intervention with usual care for children presenting to general practitioner practices with RTI and acute cough. The trial aims to recruit at least 300 children between October 2014 and April 2015, in a single area in South West England. Following informed consent, demographic information will be recorded, and symptoms and signs measured. Parents/carers of recruited children will be followed up on a weekly basis to establish symptom duration, resource use and cost of the illness to the parent until the child's cough has resolved or up to 8 weeks, whichever occurs earlier. A review of medical notes, including clinical history, primary care reconsultations and hospitalisations will be undertaken 2 months after recruitment. The trial feasibility will be assessed by: determining acceptability of the intervention to clinicians and parent/carers; quantifying differential recruitment and follow-up; determining intervention fidelity; the success in gathering the data necessary to conduct a cost-effectiveness analysis; and collecting data about antibiotic prescribing rates to inform the sample size needed for a fully powered RCT.

**Ethics and dissemination:**

The study was approved by the North West—Haydock Research Ethics Committee, UK (reference number: 14/NW/1034). The findings from this feasibility trial will be disseminated through research conferences and peer-reviewed journals.

**Trial registration number:**

ISRCTN23547970.

## Introduction

Children with respiratory tract infections (RTIs) are the single most frequent user group of primary healthcare resources, and acute cough is the most common symptom.^1^ The majority of RTIs in children are self-limiting and present a minor threat to the child's health. However, these cause a significant disruption to family life and are extremely costly for service providers, parents and employers.^2^ Despite evidence of their effectiveness in self-limiting conditions,^3^ clinicians frequently prescribe antibiotics for RTIs.^4^ The current scale of prescribing for self-limiting conditions, particularly for children, is a significant problem for the UK's National Health Service (NHS) and is associated with increased care-seeking behaviour for minor illnesses,^5^ as well as antimicrobial resistance.^6^ While the inappropriate use of antibiotics and antimicrobial resistance is now at the top of the agenda for England's Chief Medical Officer and the National Institute for Health Research (NIHR),^6^ primary care clinicians^7^ and the research community^4^ have called for the development of a sound evidence base to reduce uncertainty around differentiating children who would benefit from antibiotics from those who would not.

Although the majority of RTI cases will resolve on their own, a small proportion of children will develop severe symptoms requiring hospitalisation.^8^ Uncertainty regarding which children with RTIs are at risk of poor outcome is an important driver of both prescribing behaviour^9^ and parent consultations^10^ in primary care. Clinicians report that they will often prescribe antibiotics ‘just in case’, if they feel uncertain about social, health or legal outcomes.^11^ A perceived pressure or expectation of antibiotics by the parents of children with RTIs has also been cited by clinicians as a major contributing factor towards overprescription of antibiotics.^12^ ^13^ However, many parents have a no treatment preference when they consult for an acutely unwell child.^11^ There is poor agreement between parental expectation and clinician-perceived expectations,^13^ and clinicians can interpret several different parental communication behaviours (such as asking for information at the end of the consultation) as an expectation or pressure for antibiotics.^12^ Parents also report that they leave consultations feeling they have insufficient information about their child's illness.^5^ ^14^ ^15^

Changes in practice are needed to improve health outcomes for children. This can be achieved by identifying children for whom antibiotics are not needed, and by providing information regarding the symptoms denoting poor prognosis about which the parents should be vigilant. Clinicians have requested clear evidence-based information to reduce uncertainty around clinical prognosis of children with RTIs to support their treatment decisions in primary care.^7^ Parents have stated that they want clear information to enable them to manage their child's illness.^16^ ^17^ Indeed, parents of children with RTIs are seeking information and reassurance more than antibiotics.^10^ ^14^ Examples of such information have been shown to reduce reconsultations^18^ and antibiotic use^6^ in adults with RTI. If given positively, it may also improve consultation satisfaction.^19^ The evidence base indicates that the most effective interventions to reduce rates of antibiotic prescriptions target both parents and clinicians during consultations^20^ and provide evidence-based prescribing computer prompts.^21^ ^22^

The CHICO (Children's Cough) study is the final element of the TARGET Programme, which consists of four work streams: (1) systematic reviews,^11^ ^12^ ^21^ ^23^ ^24^ (2) qualitative studies,^10^ ^14^ ^25^ (3) a large cohort study involving 8394 children^26^ and (4) synthesising the evidence for the generation and evaluation of a complex intervention. Systematic reviews evaluated the evidence for the effectiveness of existing interventions aimed at reducing unnecessary antibiotic prescribing in primary care,^24^ natural history of RTIs in children,^23^ parent and clinician views of antibiotic prescribing,^11^ and how communication within the consultation influenced treatment decision-making.^12^ The qualitative studies investigated the views, behaviours and needs of both clinicians and parents when managing RTI in children. The cohort study generated a clinical prediction rule to determine which children with RTIs in primary care are more likely to be hospitalised. Findings from across the TARGET Programme were synthesised to develop a complex intervention designed to reduce antibiotic prescribing. The central aim of this trial is to assess the complex intervention developed in the TARGET Programme, and to understand whether it is feasible to conduct a larger trial. The aim of the full trial would be to establish whether a complex behavioural web-based intervention can contribute to the reduction in overall quantity of antibiotics prescribed and consumed, as well as improve the consultation experience for parents/carers.

## Methods and analysis

The CHICO trial is designed as a single-centre feasibility practice-cluster randomised controlled trial comparing a web-based behavioural intervention with usual care for children presenting to primary care practices with RTI and acute cough. The trial protocol was devised according to the SPIRIT guidelines for randomised controlled trials.^27^ The trial aims to recruit between 300 and 500 children in primary care sites between October 2014 and the end of April 2015, in a single area in South West England.

### Developing the intervention

Findings from across the TARGET Programme were synthesised into a logic model developed using the structure of Greene and Kreuter's Precede-Proceed model for health promotion.^28^ ^29^ Key findings from each component of the TARGET Programme were used to highlight the evidence for social, behavioural and environmental factors shown to influence clinicians’ decisions to prescribe. Development of the intervention was iterative. The parent advisory group (PAG) and clinician advisory group (CAG) provided comments and suggestions about the format of the intervention and associated materials in the early stage and final stage of development. The feedback was reviewed by the team leading the development of the intervention and modifications were made where possible.

The best evidence available suggested that this should focus on providing clinicians with accurate information about average symptom trajectories, reducing clinical uncertainty about complications that could lead to hospitalisation, improve parent-clinician communication about parent concerns, and should be followed with the provision of individualised child-focused information.

The intervention was designed to be a tool for the clinician to use within the child's RTI face-to-face consultation only. A web-based interface allows for a single intervention to be delivered and comprises four components:

1. Structured symptom and sign collection: By using a within-consultation web-based tool, clinicians will be asked to record the clinical condition of the child.
2. Elicitation of parent concerns: Evidence suggests that miscommunication between clinicians and parents drives clinicians to prescribe antibiotics, where requests for medication evaluations or information are misconstrued as requests for treatment. By explicitly eliciting parents’ concerns, we aim to reduce the likelihood of miscommunication.
3. A clinical prediction rule: To address the issue of the clinician's uncertainty about severity of the child's illness, we will use the clinical profile of the child using their demographic information and clinical presentation during the consultation. The objective is to provide an assessment of the risk that the child will be hospitalised within 30 days postrecruitment.
4. Enhanced treatment options: We provide treatment recommendations, highlighting no or delayed prescription strategies for children with low risks of hospitalisation, and a child health information booklet. The recorded clinical condition and parent concerns are used to produce a personalised information booklet that explains the best home-care strategies for each symptom, responds to parent concerns with further information, provides information about the natural history of common symptoms and provides standard safety netting information. The aim of this booklet is to not only provide more information to parents, but also to provide clinicians with a concrete treatment action other than prescribing.

### Outcome measures

The main feasibility outcome measures will be:

- The acceptability of the intervention to practising primary care clinicians and parents/carers;
- Recruitment at the practice and participant levels, attrition and retention of both clinicians and participants to the trial;
- Parents/carers and clinicians views on the intervention experience;
- To ascertain the levels of antibiotic prescribing in the intervention and control groups (including CIs for these levels) so as to inform a power calculation for a full trial.

Additional outcomes will include trial fidelity measures, and clinical and trial data collection variables to inform the primary and secondary outcomes of the larger trial. These will include, but are not limited to:

- Whether antibiotics were prescribed, either immediately or delayed, at the conclusion of the consultation (baseline) and whether the prescribing was in line with the intervention's recommendations.
- Referrals to secondary care at the time of the consultation and admission to secondary care or to the emergency department (ED) in the 30 days postrecruitment.
- Antibiotic consumption within 30 days postrecruitment for both baseline consultation and any reconsultations; whether any antibiotics were prescribed after consultation and prior to illness resolution.
- Symptom duration—time from when the child presented to primary care with cough and was recruited to trial to when the child's symptoms were resolved.
- Reconsultations (due to illness deterioration) to primary care in the 30 days postrecruitment.
- Wellness as defined in the CHU-9D questionnaire for those children aged 5–11 years only; resources required to care for children with RTI from the viewpoint of the NHS, families and lost productivity, and the completeness of this resource-use data.
- Use and acceptability of the intervention will be investigated by:
  Investigating frequency with which intervention is initiated;
  Establishing the time the intervention takes and the rate of any abandoned consultations;
  Whether clinicians revise their treatment outcome decision (and the number of times) in the consultation;
  Qualitative interviews with parents from the intervention arm and clinicians from both arms of the trial.

### Sample size

As this is a feasibility trial, we are interested in the recruitment rate in the intervention and control groups to inform the design of a larger main trial. We will monitor differential recruitment to inform whether a 1:1 recruitment strategy should be used for the full trial, and whether we need to provide more training/clarity to general practitioners (GPs) regarding the inclusion and exclusion criteria.

Recruitment rates from the TARGET cohort study, using Bristol practices between October and April, suggest around 10 patients per practice were recruited (mean=11.2 (95% CI 8.9 to 13.5)). To ensure we will have sufficient participants for our process measures, at least 15 practices per arm of the trial will be recruited and randomised. This number of practices takes into consideration the large variability we have previously observed in recruitment between practices and the potential to assess differential recruitment between the arms of the trial. Practices will be expected to recruit at least 10 participants per practice in 7 months, enabling a minimum of 300 children to enter the (feasibility) trial. This should be sufficient to calculate sufficiently precise estimates of the feasibility outcomes listed above.

### Practice recruitment

General practices will be invited to participate in the trial from locations in Bristol and the surrounding areas via the local primary care Clinical Research Network (CRN). The recruiting clinicians will attempt to recruit all eligible patients during the 7-month recruitment period. Recruiting clinicians will record online if participants declined to participate or were missed and the reasons for these labelled as ‘patient not recruited’.

A large geographical area of practices will be invited to participate to avoid saturation of research studies in some practices, and to maximise the generalisability of the sample of children.

### Randomisation process

The randomisation process will be a one-off randomisation of each recruited primary care site (general practice) occurring before the start of study recruitment. Given that general practice consultation and antibiotic prescribing rates tend to be higher in some areas than others, the randomisation process will be stratified to achieve balance with respect to: total number of children in the age range registered at the practice (list size); and proportion of children's amoxicillin antibiotic prescriptions per child in the practice, as a proxy measure of antibiotic prescribing for the practice (prescribing rate).

Owing to the nature of the intervention delivery, it will not be possible to blind practices to their allocation to either control or intervention group. Nevertheless, the explanation prior to randomisation emphasises optimising RTI management rather than reducing antibiotic prescribing, and is the same for both groups. At the individual level, the parents will be invited to participate in the feasibility study knowing this is a trial testing two approaches to RTI management, but will not be aware whether they are in the intervention or control arm. Furthermore, the research staff reviewing participants' primary care medical record, assessing outcomes and conducting the analysis will not know to which group participants were allocated. Balance across the arms for individual characteristics will be monitored carefully by the Data Monitoring Committee (DMC) to ensure differential recruitment is minimised as much as possible. In order to minimise bias in the trial^30^ and in data collection, trial processes were reviewed prior to the start of practice and participant recruitment to assess internal and external validity measures. Processes have been put in place specifically to tackle the known issue of selection bias in cluster trials such as emphasising that clinicians recruit all eligible children and requesting that they systematically record reasons for not recruiting children.^30^

### Participant recruitment

Practices will publicise the study in a number of ways—for example, on the practice website or in their newsletter. Families telephoning for an appointment and attending primary care sites with their potentially eligible children will be invited to participate in the trial either by the reception staff or by their clinicians. Eligibility will be checked and informed consent will be obtained for participants from the parent or legal guardian presenting to both intervention and control group practices. Participants have at least 24 h to decide if they wish to participate as this will be checked at the first follow-up contact. Participants will leave the consultation with an information pack containing the participant information sheet, copy of their consent form, and follow-up questionnaire. No incentives will be provided to encourage participants to enter the study. A £5 shopping voucher will be provided to families by way of a ‘thank you’ for completion of the follow-up data collection.

### Participants

Children will be included if they are between 3** **months and 12** **years of age, and present with a RTI with cough of no more than 28** **days duration prior to consultation. They will be eligible if they present with illnesses such as asthma, epilepsy or diabetes, and RTI, including infective exacerbation of asthma, as well as children who require same day hospital assessment or admission. Children will be excluded if they: present with acute, non-infective exacerbation of asthma; present with RTI without cough or cough of more than 28 days; are considered to have a high risk of serious infection (eg, immunocompromised, cystic fibrosis, splenectomy); are temporarily registered with the NHS primary care site (general practice, walk-in centre, GP out of hours centre or polyclinic) and are likely to be unregistered/non-resident within a month; have parent/carers who are unable or unwilling to assist with the study; are already recruited to the CHICO trial.

### Data collection

Baseline data will be collected from the participants and parents/carers by both control and intervention clinicians during a routine clinical consultation. The clinicians will enter the child's demographic characteristics, carer-reported symptoms, clinician-measured signs and clinical outcomes of the consultation onto a secure web-based database. The intervention group will also have the facilities to record parental concerns on the web-based database. Information about user interaction with the web-based system will be collected for control and intervention versions of the site. Exposure to the site will be assessed by measuring the number of times the clinicians use the web-based intervention and the amount of time spent on each page of the site.

Follow-up data will be collected from parents/carers weekly (by phone or online) until the child's symptoms are resolved, or for 8 weeks if the parent informs the team that the cough has not resolved. The information collected will include whether the child's symptoms have resolved, antibiotic consumption, any further contact with health professionals, quality of life data, relevant out-of-pocket expenditure and associated time off work. The aim of the health economics component of the study will be to inform the design of a full economic evaluation alongside the larger trial for assessing the intervention in terms of value for money.

Parents/carers in the intervention group and clinicians from both groups will be invited to participate in semistructured interviews to explore their views, experiences and acceptability of the intervention. Interviews with parents/carers will be conducted either in the week following recruitment (in order to facilitate recall of the consultation experience) or after their child has recovered to reflect on the whole follow-up data collection process. Purposive sampling will be used to include a maximum variation sample of parents/carers, including those with a range of ages of children and ethnicity; participants will be selected from areas of high and low social-economic deprivation, and with a range of illness severity scores and treatment outcomes. We will interview one clinician from each participating practice and up to 30 parents/carers in total to explore views and experiences of the intervention. These interviews will help us gain understanding of the barriers and facilitators of the intervention implementation to inform the design of a future, larger trial. Clinicians who agree to participate in interviews will be reimbursed financially for their time. A £5 shopping voucher will be provided to families as a ‘thank you’ for participating in the interviews.

We will conduct a primary care medical record review at the recruiting practices to collect data relating to the 30 days following the recruitment consultation. This will include information about the time taken for the recruiting consultation, and RTI-related antibiotic prescriptions, reconsultations and hospitalisations. The medical record review will also record consultations and related antibiotic prescriptions for RTI in the year prior to recruitment. The review will be completed by a member of the research team who will be trained in the medical notes system for the practice. All data will be recorded on the secure web-based database and anonymised. These data can also be used to verify the data collected by the clinicians at recruitment and reported by the parent/carers at the follow-up and this will help to inform whether the medical record review is a robust mechanism for the larger trial.

### Data analysis procedure

The main statistical analyses will be carried out according to the study analysis plan. Descriptive statistics (means, SDs and non-parametric measures, where appropriate) will be used to describe the characteristics of the parents/carers, children and recruiting clinicians from the control and intervention group practices. To help address our outcomes, we will: (1) investigate the post-test probability of hospitalisation thresholds at which different clinicians choose to prescribe immediate or delayed antibiotics; (2) scrutinise between-group differences in prescribing rates and calculate 95% CIs to inform the sample size of a larger trial; (3) scrutinise antibiotic prescribing rates postconsultation in both arms to offset transference of prescribing at a later date; (4) collect information on reconsultations, ED visits and hospitalisations during follow-up from the parents, which will be compared with the information collected at the medical record review to assess whether this latter method will be robust enough for the larger trial; (5) estimate the intraclass correlations by practice of the outcome measurements (although we appreciate the small number of practices will limit our conclusions); (6) study the variability of the size of clusters from each practice to help inform the inflation factor for our sample size; (7) conduct intervention group logistic regression to look at differences in parental concerns about hospitalisation between the very low-risk group and the normal-risk and high-risk groups, which will help inform whether we have comprehensively addressed all concerns and weighted the advice accordingly. All quantitative data will be analysed using Stata V.12.0.

Interviews will be transcribed and anonymised. Analysis of qualitative data will begin shortly after data collection starts, and will be ongoing and iterative. Analysis will inform further data collection, and the analytic insights from data gathered in earlier interviews will help identify any changes that need to be made to the topic guides during later interviews. Qualitative analysis of the transcripts will follow recognised thematic analysis procedures using NVivo software. Thematic analysis, utilising a data-driven inductive approach, will be used to scrutinise the data in order to identify and analyse patterns and themes of particular salience for participants and across the data set. Transcripts will be coded and global themes developed from the codes. Two researchers will code the transcripts, and any differences will be discussed and resolved within the research team in order to achieve a coding consensus and to ensure robust analysis.

Health economic analysis will be exploratory. We will provide descriptive statistics on resource use and cost by category of resource and by randomisation group in order to identify important cost drivers. We will also investigate the level and nature of missing data to assess the methods of data collection and inform how best to carry this out in the larger trial. We will review the appropriateness of different outcomes with which to compare costs.

The fidelity measures will be explored to respond to the detailed plans for the intervention content and implementation. This will be achieved by constructing an ‘intervention map’ that will specify all the behavioural components that we wish to change. Where possible, parallel process measures will be developed to establish the extent to which processes or outcomes have been modified. These are likely to include measures for: (1) adherence to the intervention delivery, (2) exposure to the intervention, assessed by measuring the number of times the clinicians use the web-based intervention, the amount of time spent reading the background information as well as an assessment of the quality of the intervention delivered, and (3) differentiation between intervention and control groups in terms of recruitment levels, attrition rates and disease severity.

### Patient and public involvement

This proposal has been developed collaboratively with our PAG, and their comments and suggestions about the format of the intervention and parent/carer materials have informed for both the intervention and the final design of the feasibility study. The PAG will meet throughout the study, twice per year, allowing the investigators to report on progress of the study and discuss issues that arise during the study. PAG members will input in all the material for parents/carers as and when these are developed, including the patient information sheet, intervention materials and the topic guide for interviews. We have also formed a CAG, involving GPs and practice nurses, to assist with developing and refining the intervention. They will meet once in person and then contribute by Skype or email to refine GP/nurse information and intervention delivery. We will work closely with our advisory groups, particularly during the first 9 months of this work package.

## Ethics and dissemination

### Burden

As the feasibility trial is assessing a behavioural intervention and participants are attending for a routine consultation with a clinician, there are no risks to the participants taking part in this study. Children will not be randomised to receive a medication or intervention, and no treatment will be withheld. Further clinical management will be decided on by the patient's clinician, as it is the choice of the recruiting clinician as to whether the participants are treated or not for their cough.

The main burden will be the additional time associated with participating in the study. This has been reduced to a minimum by only asking some short concise questions during their routine consultation, and questions about the child’s recovery, treatment and healthcare resources at follow-up.

The intervention itself will be assessed as to whether the components of the intervention could potentially increase hospitalisations by virtue of reducing antibiotic prescribing, and so families will be asked weekly whether their child has been hospitalised in the preceding 7 days via the weekly phone call.

### Adverse events and serious adverse events

Any unexpected adverse events (AEs) defined as ‘any untoward medical occurrence in a trial participant’ and serious AEs (SAEs)—defined below—will be monitored by the trial team and reviewed at monthly management group meetings.

From our earlier work, we anticipate that approximately 1% (2–4 children) of children presenting with RTI will be hospitalised. We will ask recruiting practices to inform the trial coordinator of any unexpected SAEs that occur during the child's participation in the trial.

The principal investigator and trial coordinator will assess the nature of the SAE for seriousness, causality and expectedness. Following the initial report, follow-up data may be requested by the trial coordinator. All reports will be submitted to the DMC for scrutiny of our ongoing findings. Where the SAE is both related and unexpected, the trial coordinator will notify the main REC, the sponsor of the trial, and its research governance office within 15 days of receiving notification of the SAE.

As one of the outcomes for the trial is hospitalisation, we do expect some participants to be admitted to hospital due to a deterioration of their underlying illness, for example, pneumonia, empyema and deteriorating bronchiolitis. At follow-up, parents/carers will be routinely asked if they have reconsulted for their child's illness and if so, where this was done. In addition, whether the child has been hospitalised will also be asked. Once a child has been identified as hospitalised, either in the ED or on a ward, the standard SAE process will be followed as described above. All expected SAEs will be reported as part of the outcome to the trial.

### Definition of a SAE

This is any untoward and unexpected medical occurrence or effect that: results in death; is life-threatening (refers to an event during which the participant was at risk of death at the time of the event, and not to an event that might have caused death had it been more severe in nature); requires hospitalisation or prolongation of existing hospitalisation; or results in persistent/significant disability or incapacity.

### Study sponsorship

The University of Bristol will act as sponsor for study. Delegated responsibilities will be assigned to the Universities and NHS trusts taking part in this study. The study is open to inspection and audit by the University of Bristol under its remit as sponsor.

### Dissemination

The outputs from this research will comply with the TARGET Programme's publication policy. All results will be described in the Programme's final report and as papers in peer-reviewed journals. Papers will be produced to describe the findings from this feasibility study, and results will be presented at national and international research conferences. A summary of the findings will be sent to participating practices (and this will be available to their patients) on completion of the CHICO study.
